# Long-Term Treatment of Cuban Policosanol Attenuates Abnormal Oxidative Stress and Inflammatory Response via Amyloid Plaques Reduction in 5xFAD Mice

**DOI:** 10.3390/antiox10081321

**Published:** 2021-08-23

**Authors:** Jin-Ho Kim, Dong-Kyun Lim, Yoo-Hun Suh, Keun-A Chang

**Affiliations:** 1 Department of Health Sciences and Technology, Gachon Advanced Insiue for Healh Sciences & Technology (GAIHST), Gachon University, Incheon 21999, Korea; qpdlzhfl@gachon.ac.kr; 2 Neuroscience Research Institute, Gachon University, Incheon 21565, Korea; bydongkyun@gmail.com (D.-K.L.); yhsuh@snu.ac.kr (Y.-H.S.); 3 Department of Pharmacology, College of Medicine, Seoul National University, Seoul 03080, Korea; 4 Department of Pharmacology, College of Medicine, Gachon University, Incheon 21999, Korea

**Keywords:** policosanol, Alzheimer’s disease, 5xFAD mice, antioxidant effects, anti-inflammation, memory improvement

## Abstract

Alzheimer’s disease (AD) is a progressive neurodegenerative disorder resulting in cognitive decline or dementia, the number of patients with AD is continuously increasing. Although a lot of great progress has been made in research and development of AD therapeutics, there is no fundamental cure for this disease yet. This study demonstrated the memory-improving effects of Cuban policosanol (PCO) in 5xFAD mice, which is an animal model of AD. Following 4-months of treatment with PCO in 5xFAD mice, we found that the number of amyloid plaques decreased in the brain compared to the vehicle-treated 5xFAD mice. Long-term PCO treatment in 5xFAD mice resulted in the reduction of gliosis and abnormal inflammatory cytokines level (interleukin [IL]-1β, IL-6, and tumor necrosis factor [TNF]-α) in the cortex and hippocampus. Levels of lipid peroxide (4-hydroxynonenal [4-HNE]) and superoxide dismutase (SOD1 and SOD2) levels were also recoverd in the brains of PCO-treated 5xFAD mice. Notably, PCO administration reduced memory deficits in the passive avoidance test, as well as synaptic loss (PSD-95, synaptophysin) in 5xFAD mice. Collectively, we identified the potential effects of PCO as a useful supplement to delay or prevent AD progression by inhibiting the formation of Aβ plaques in the brain.

## 1. Introduction

Alzheimer’s disease (AD) is a progressive neurodegenerative disease associated with cognitive decline. Recent investigations indicate that the number of dementia patients is projected to increase by more than 131 million worldwide by 2050, together with the cost of dementia [1]. AD has a neuropathological hallmark consisting of extracellular amyloid plaque deposition composed of amyloid beta (Aβ) peptide and intracellular neurofibrillary tangles (NFT) containing hyperphosphorylated tau [2,3,4]. Amyloid plaques and NFT are mainly deposited in the brain, such as the hippocampus, entorhinal cortex, and basal forebrain, which influence learning, memory, and emotional behavior [5]. Eventually, these neuropathologies result in damage and destruction of the synapses that mediate memory and cognition [6]. Although research on the treatment of AD is continuously progressing, currently no clear preventive or therapeutic drugs exist.

Although Aβ_40_ (~80–90%) is most abundant, Aβ_42_ (~5–10%) has a higher aggregation propensity, and mainly consists of the amyloid plaques and is assigned to be toxic [7,8,9]. Many studies reported that Aβ aggregation triggered gliosis, activated glial cells, and induced neuronal loss, as well as synaptic degeneration [10,11]. Specifically, amyloid plaques induced gliosis, and these reactive astrocytes and activated microglia located near the site of amyloid plaques in the brain including the cortex and hippocampus [12,13]. The activation of glial cells released the AD-related cytokines such as IL-1β, IL-6, and TNF-α, as well as ROS generation [14,15,16]. As a result, the abnormal inflammatory immune response is related to neuronal cell death and damage.

Since the brain is especially vulnerable to oxidative stress, which is defined as an imbalance of increased free radicals or decreased antioxidant defense mechanisms, oxidative stress is an important pathogenic factor occurring in the early stage of AD [17,18]. Many studies have reported that Aβ can induce increased production of ROS and damage the mitochondria, which is accompanied by abnormal mitochondrial structure and impaired mitochondrial respiration [9,19,20]. Lipid peroxidation is the most prominent and pronounced of neurodegenerative changes. Specifically, increased levels of 4-hydroxynonenal (4-HNE), a marker of lipid peroxidation, have been detected in the brains of patients with AD [21]. Free or protein-bound 4-HNE induced by Aβ increases neuronal vulnerability to neurotoxicity and impairs the Ca^2+^ concentration [22]. Furthermore, the altered expression level of superoxide dismutase (SOD), which initially acts as an antioxidant in the defense against ROS, has been detected in patients with AD and animal models [23,24]. These abnormal regulations, such as excessive ROS and impaired antioxidant system, aggravate AD progression.

Cuban policosanol (PCO) is a mixture of alcohols separated and purified from sugar cane (*Saccharum officinarum* L). Especially, PCO reported safety as well as the effect on dyslipidemia in the meta-analysis [25]. A previous report suggested that PCO consumption by patients with type II hypercholesterolemia, type 2 diabetics with hypercholesterolemia, and combined hypercholesterolemia improved the lipid-related risk factors such as low-density lipoprotein (LDL) oxidation in cardiovascular disease [26]. Moreover, in a human study in Korea [27,28], short-term (12 weeks) and long-term (24 weeks) consumption of PCO resulted in decreased blood pressure and serum total cholesterol with the elevation of high-density lipoprotein-cholesterol (HDL-C) in a dose-dependent manner. In addition to its lipid-lowering effect, PCO showed antioxidant and anti-glycation effects to enhance HDL functionality [29].

Since abnormalities in lipid metabolism are important risk factors in AD pathology, PCO treatment may be an effective substrate in the management of AD. In the current study, we aimed to analyze the effects of long-term policosanol consumption on memory impairment in 5xFAD mice, which is an animal model of AD.

## 2. Materials and Methods

### 2.1. Animals

Two-month-old male transgenic 5xFAD (B6SJL) mice and wild-type litter-mate mice as controls were used in this study. The 5xFAD mice express APP [Swedish (K670N/M671L), Florida (I716V), and London (V717I) mutations] and PS1 (M146L/L286V mutations) transgenes. Mice were the progeny of male hemizygous 5xFAD mice selected from existing in-house animals and female wild-type F1 hybrid mice obtained from JAX Laboratories (Bar Harbor, ME, USA). The temperature and humidity of the breeding room were automatically maintained at 22 ± 2 °C and 50 ± 10%, respectively. A 12L:12D photoperiod was provided, and food and water were provided *ad libitum* during the acclimation period in cages. All the animal experiments were approved by the Institutional Animal Care and Use Committee of the Lee Gil Ya Cancer and Diabetes Institute, Gachon University (LCDI-2018-0145).

### 2.2. Policosanol Treatment

**Figure 1:**
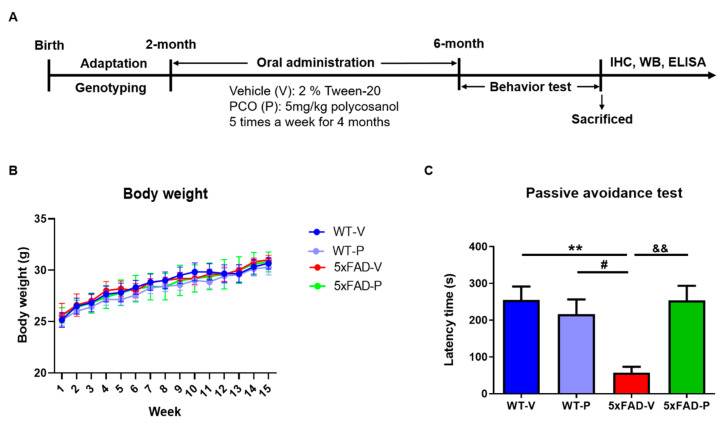
The policosanol effect on cognitive dysfunction and pathophysiology of Alzheimer’s disease in 5xFAD mice. (**A**) The scheme of the experiment procedure. After treatment with policosanol by oral injection (5 mg/kg) for 4 months and then performing the behavior test to assess the effect of cognitive behavior. (**B**) The bodyweight of mice was measured weekly. (**C**) Policosanol treatment for 4 months increased latency time in the 6 months aged 5xFAD mice. Values are expressed as the mean ± SEM (*n* = 5~6 per group). ** *p* < 0.01 vs. WT-V group, ^#^ *p* < 0.05 vs. WT-P, ^&&^ *p* < 0.01 vs. 5xFAD-V group. Statistical analysis between the four groups was performed using the one-way ANOVA, followed by Turkey’s post hoc test. WT-V: Vehicle-treated wild-type mice; WT-P: Policosanol-treated wild-type mice; 5xFAD-V: Vehicle-treated 5xFAD mice; 5xFAD-P: Policosanol-treated 5xFAD mice.

The policosanol raw material was obtained from Rainbow & Nature Pty, Ltd. (Thornleigh, NSW, Australia). The policosanol comprised several alcohol chains of various lengths, and over 90% of the policosanol content was higher in the aliphatic wax alcohols. Policosanol formulations contain aliphatic alcohols in the following proportions [27]: 1-tetracosanol (C_24_H_49_OH, 0.1–20 mg/g), 1-nonacosanol (C_29_H_59_OH, 1.0–20.0 mg/g), 1-heptacosanol (C_27_H_55_OH, 1.0–30.0 mg/g), 1-tetratriacontanol (C_34_H_69_OH, 1.0–50.0 mg/g), 1-hexacosanol (C_26_H_53_OH, 30.0–100.0 mg/g), 1-dotriacontanol (C_32_H_65_OH, 50.0–100.0 mg/g), 1-triacontanol (C_30_H_61_OH, 100.0–150.0), and 1-octacosanol (C_28_H_57_OH, 600.0–700.0 mg/g). Policosanol (5 mg/kg) suspended in 2% Tween 20 (DUCHEFA, Haarlem, Netherlands) was administered orally by gastric gavage to 5xFAD or littermate wild-type mice beginning at 2-months of age. Saline containing 2% Tween 20 was used as the vehicle in the 5xFAD or wild-type mice. Daily administration persisted for 4 months, 5 times per week (Figure 1A). A suspension of policosanol was freshly prepared before use. In each experimental group, 2-months old 5xFAD or WT mice were used, which comprised 5–6 male mice in the following groups: Vehicle-treated wild-type mice (WT-V), policosanol-treated wild-type mice (WT-P), vehicle-treated 5xFAD mice (5xFAD-V), and policosanol-treated 5xFAD mice (5xFAD-P) (Figure 1A).

### 2.3. Passive Avoidance Test

The passive avoidance test was performed for three continuous days using a passive avoidance apparatus (Gemini Passive Avoidance System; San Diego Instruments, San Diego, CA, USA). It had two adjacent chambers that were bright and dark, and the chambers were connected by a remote operational gate. The bright chamber was illuminated by a 6 W LED light. During the test, the mice were placed in a bright room. On the first day, we allowed the mice to freely explore the chambers and trained them to enter the dark chamber when the gate was open. The next day, the mice were exposed to electric shock on their feet (2 mA for 2 s) after entering the dark chamber. By 24 h after the retention test, the time duration from the placement of the animal in the bright chamber until their entrance to the dark chamber was measured as the step-through latency time, which indicates their memory retrieval. All the daily procedures were performed between 9:00 and 14:00.

### 2.4. Histological Preparation

#### 2.4.1. Tissue Preparation

Under anesthetizing with a mixture of Zoletil (16.7 mg/kg) and Rompun (15.5 mg/kg), the mouse’s brain was extracted. The hemisphere of each mouse brain was fixed in 4% paraformaldehyde at 4 °C for 24 h and then was dehydrated in a 30% sucrose solution for 3 days. The dehydrated tissues were frozen in molds filled with optimal cutting temperature compounds (Sakura, Osaka, Japan). After frozen tissues were cut at a thickness of 22 μm using the cryomicrotome (Cryotome, Thermo Electron Corporation, Waltham, MA, USA), tissues were placed in a cryoprotectant solution (ethylene 30% and glycerol 30% in PBS), and stored at 4 °C.

#### 2.4.2. Immunohistochemistry and Quantification

To determine the effects of policosanol on microglial and astrocyte activation in 5xFAD mice, immunohistochemical analysis was performed as previously described [30]. Briefly, cryostat brain sections were washed in PBS-T (0.3% Triton X-100 in PBS), and the slices were blocked in PBS-T containing 0.5% Bovine serum albumin (BSA) and 3% normal goat serum at room temperature for 30 min. Sections were then incubated with primary antibody overnight at 4 °C in PBS-T solution (glial fibrillary acidic protein [GFAP], 1:500, DAKO; Iba1, 1:500, Novus Biologicals, Centennial, CO, USA). The next day, the following incubation with fluorescent secondary antibody for 1 h at room temperature, and 4′,6-diamidino-2-phenylindole (DAPI) was counterstaining. Images were taken using a Zeiss AxioImager (White Plains, NY, USA) Z1 microscope at 100× magnification. To construct complete images of the hippocampus, separate images taken in the CA1, CA3, and dentate gyrus (DG) were combined according to their correlated parts of the images. Once the region of interests (ROIs) were defined, Alexa Fluro 488 (green) or Alexa Fluro 555 (red) signal was used to measure the fluorescence intensity or the percent of a red signal within each ROI (*n* = 3 per animal).

#### 2.4.3. Amyloid Plaque Staining and Quantification

To stain amyloid plaques, thioflavin S stain was applied to the sections for 10 min at room temperature. Finally, the dyed tissues were mounted on glass slides and imaged for further analysis. Images were taken using a Zeiss AxioImager Z1 microscope at 100× magnification. To construct complete images of the hippocampus, separate images taken in CA1, CA3, and dentate gyrus were combined according to their correlated parts of the images. The number of amyloid plaques in the brain sections was counted manually using the fluorescent signal by thioflavin S in the entorhinal cortex, prefrontal cortex, and hippocampus. We used the Image J software (V1.4.3.67, National Institute of Health, Bethesda, MD, USA) for manual counting of the plaques.

### 2.5. Western Blot

The cortex and hippocampus of the mice were lysed with radioimmunoprecipitation assay (RIPA) buffer (150 mM NaCl, 1% NP-40, 0.5% sodium deoxycholate, 0.1% SDS, 50 mM Tris, pH 8.0) containing protease inhibitors (Roche Applied Science, Mannheim, Germany) and a cocktail of phosphatase inhibitors (Sigma-Aldrich, St. Louis, MO, USA) in ice for 30 min. The lysates were centrifuged at 13,000 rpm for 10 min at 4 °C. The samples were quantified using the Bradford assay (Bio-Rad Laboratories, Inc., Hercules, CA, USA), loaded onto an 8 or 15% sodium dodecyl sulfate-polyacrylamide gel electrophoresis (SDS-PAGE). Proteins were transferred onto a polyvinylidene difluoride (PVDF) membrane (Merck, Kenilworth, NJ, USA). After blocking the transferred membrane with blocking buffer (3% BSA in TBS-T) at room temperature for 1 h, the membrane was incubated with the appropriate primary antibody (4-HNE, 1:1000; R&D: SOD1, 1:3000; SOD2, 1:3000; NOS2 [iNOS], 1:1000; Santa Cruz (Dallas, TX, USA) Synaptophysin, 1:3000; PSD-95, 1:3000; β-actin, 1:3000; Santa Cruz) overnight at 4 °C. After washing, the following incubation with secondary antibody for 1 h at room temperature was done. Protein bands were detected using Enhanced Peroxidase Detection (PicoEPD) enhanced chemiluminescent (ECL) (ELPISBIO, Daejeon, Korea) or Immobilon Western Chemiluminescent HRP Substrate (Millipore, Burlington, MA, USA) and BLUE X-ray film (AGFA, Mortsel, Belgium). Quantification of the bands was performed using the ImageJ software v1.4.3.67.

### 2.6. Enzyme-Linked Immunosorbent Assay

Enzyme-linked immunosorbent assays (ELISAs) were used to quantify the levels of Aβ_1–__42_, IL-1β, IL-6, and TNF-α. To create the total lysate, the cortical tissues of the mice were weighed and homogenized in RIPA buffer containing a cocktail of protease inhibitors (Roche Science, Mannheim, Germany) and a cocktail of phosphatase inhibitors (Sigma-Aldrich, St. Louis, MO, USA). Brain tissue homogenates were analyzed using the following ELISA kits: Aβ_1–__42_ (KHB3544, Invitrogen, Waltham, MA, USA), IL-1β (KET6013; Abbkine, Wuhan, China), IL-6 (KET7009; Abbkine, Wuhan, China), and TNF-α (KET7015; Abbkine), according to the manufacturer’s instructions. All the experiments were analyzed in duplicate, and the mean value of the duplicate samples was calculated in all the assays. Quantification of Aβ_1–__42_ and cytokine levels was performed using a VICTOR X4 Multimode Plate Reader (PerkinElmer, Waltham, MA, USA).

### 2.7. Statistical Analysis

All the data are presented as the mean ± standard error of the mean (SEM). Statistical analysis was performed using GraphPad Prism 9.1.0 (221) software (GraphPad Software Inc., San Diego, CA, USA). One-way analysis of variance (ANOVA) was performed using the Turkey test for multiple comparisons among the groups. Statistical significance was set at *p* < 0.05.

## 3. Results

### 3.1. Long-Term Administration of PCO Recovers the Memory Deficit in 5xFAD Mice 

After the treatment of 2 months-old 5xFAD mice with PCO for 4 months, we examined the protective effect of PCO on memory deficits (Figure 1A). The bodyweight of the mice was measured every week following injection; there were no significant differences among the groups (Figure 1B). 

The passive avoidance test was performed to confirm the protective effect on memory loss after the PCO treatment. The step-through latency time of their transition from the bright room to the darkroom was measured on the last day. The 5xFAD-V group (5xFAD-V, 57.00 ± 16.24 s, Figure 1C) showed significantly decreased latency time in the light compartment compared to the wild-type mice (WT-V, 255.20 ± 16.24 s, *p* < 0.01; WT-P, 216.20 ± 39.97 s, *p* < 0.05). In addition, the 5xFAD mice treated with PCO (5xFAD-P, 253.60 ± 39.77 s, *p* < 0.01) significantly increased the latency time compared with the 5xFAD-V group.

### 3.2. PCO Treatment Decreases the Formation of Amyloid Plaques in the Cortex and Hippocampus of 5xFAD Mice

**Figure 2:**
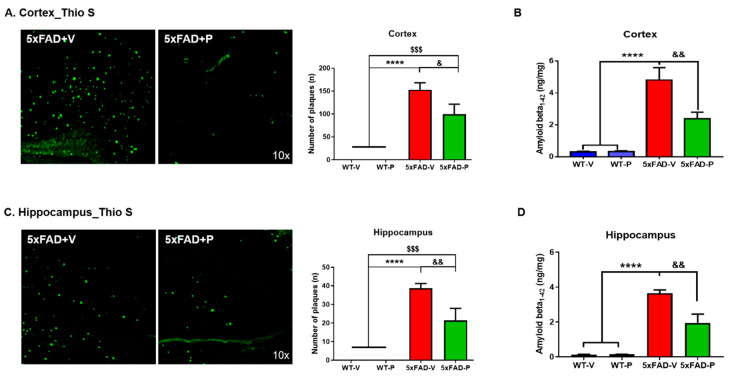
Policosanol treatment attenuated the accumulation of amyloid plaques. (**A**) The image and quantification of Thioflavin-S stain targeted the amyloid plaques in the cortex of the 5xFAD mice. The number of plaques significantly decreased in the 5xFAD-P (5 mg/kg) group compared to the 5xFAD-V group. (**B**) Amyloid beta_1–42_ in the cortex also decreased in the 5xFAD-P (5 mg/kg) group compared to the 5xFAD-V group. (**C**) The image and quantification of Thioflavin-S stain targeted the amyloid plaques in the cortex of the 5xFAD mice. The number of plaques was significantly decreased in the 5xFAD-P group compared to the 5xFAD-V group. (**D**) Amyloid beta_1–42_ in the hippocampus was also decreased in the 5xFAD-P group compared to the 5xFAD-V group. Values are expressed as the mean ± standard error of the mean (*n* = 5–6 per group). **** *p* < 0.0001, ^$$$^ *p* < 0.001 vs. compared to WT-V or WT-P group, and ^&^ *p* < 0.05, ^&&^ *p* < 0.01 vs. 5xFAD-V group. Statistical analysis between the four groups was performed using the one-way analysis of variance, followed by Turkey’s post hoc test.

To evaluate the amyloid pathological changes, we conducted two methodologies, Thioflavin-S staining for amyloid plaque load and ELISA analysis for Aβ_1–42_ protein levels in the brains of 5xFAD mice. After 4 months of daily oral administration of PCO, we observed that amyloid plaques accumulation diminished in both the cortex and the hippocampus. Interestingly, the administration of PCO (99.40 ± 22.01, *p* < 0.05) decreased the number of amyloid plaques in the cortical regions of the 5xFAD mice (57.00 ± 16.24, Figure 2A). Quantification of Aβ_1–42_ also showed that the amount of Aβ_1–42_ of the 5xFAD-P (2.38 ± 0.41 ng/mg, *p* < 0.01) mice were significantly reduced compared to the 5xFAD-V group (4.81 ± 0.78, Figure 2B).

In addition to the cortex, the amount of neuritic plaque in the hippocampus of the 5xFAD-P group (21.40 ± 2.57, *p* < 0.05) was decreased compared to that in the 5xFAD-V group (38.67 ± 2.57, Figure 2C). In the hippocampus, the Aβ_1–42_ peptide in the 5xFAD-P group (1.95 ± 0.50, *p* < 0.01) significantly decreased compared to that in the 5xFAD-V group (3.64 ± 0.20, Figure 2D). These results indicate that the PCO treatment has the effect of not only reducing the number of amyloid plaques but also reducing the protein level of Aβ_1–42_ that composes the amyloid plaques.

### 3.3. Long-Term Administration of PCO Attenuated Gliosis in the Brain of 5xFAD Mice

**Figure 3:**
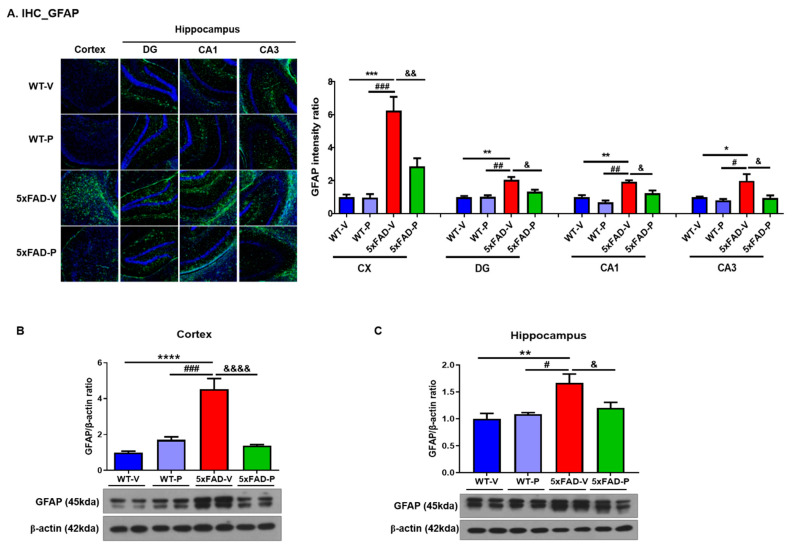
Cuban policosanol (PCO) treatment attenuated induced astrogliosis in the 5xFAD mice. (**A**) Image and quantification of glial fibrillary acidic protein (GFAP) immunofluorescence stain in the cortex and hippocampus (DG, CA1, and CA3). The GFAP expression level in the 5xFAD-P group significantly decreased compared to the 5xFAD-V group. After brain collection in the 6-months aged mice, Western blot was performed to observe the changes in the astrocytes. In both (**B**) the cortex and (**C**) hippocampus, the expression level of GFAP, which is a marker of astrocyte, in the 5xFAD treated policosanol group was reduced compared to the 5xFAD-V group. Values are expressed as the mean ± standard error of the mean (*n* = 3 per group). * *p* < 0.05, ** *p* < 0.01, *** *p* < 0.001, and **** *p* < 0.0001 vs. WT-V group, ^#^ *p* < 0.05, ^##^ *p* < 0.01, and ^###^ *p* < 0.001 vs. WT-P group, ^&^ *p* < 0.05, ^&&^ *p* < 0.01, and ^&&&&^ *p* < 0.0001 vs. 5xFAD-V group. Statistical analysis between the four groups was performed using the one-way analysis of variance, followed by Turkey’s post hoc test.

Since markedly increased levels of reactive astrocytes and microglia were present near the amyloid plaques in AD, the effects of PCO treatment on gliosis in the brains of the 5xFAD mice were analyzed. First, we performed immunohistochemistry and Western blotting with the GFAP antibody, an astrocyte marker, in the brains of all the mice groups (Figure 3). GFAP immunofluorescence staining and quantified results demonstrated that the PCO treatment reduced GFAP-reactive astrocytes in the 5xFAD-V mice (Figure 3A). The intensity ratio of astrocyte was significantly increased in the cortex and hippocampus including the DG, CA1, and CA3 in the 5xFAD-V group (cortex, 6.25 ± 0.84; DG, 2.05 ± 0.17; CA1, 1.92 ± 0.10; CA3, 1.99 ± 0.40; Figure 3A) compared to the WT-V (cortex, 1.00 ± 0.15, *p* < 0.001; DG, 1.00 ± 0.06, *p* < 0.01; CA1, 1.00 ± 0.11, *p* < 0.01; CA3, 1.00 ± 0.02, *p* < 0.05) or WT-P group (cortex, 0.97 ± 0.21, *p* < 0.001; DG, 1.01 ± 0.17, *p* < 0.01; CA1, 0.78 ± 0.12, *p* < 0.01, CA3, 0.80 ± 0.08, *p* < 0.05). Astrogliosis in the brain of the 5xFAD mice recovered by the PCO treatment (cortex, 2.854 ± 0.4988, *p* < 0.01; DG, 1.32 ± 0.12, *p* < 0.05; CA1, 1.23 ± 0.17, *p* < 0.05, CA3, 0.94 ± 0.15, *p* < 0.05). On confirmation of the protein level using Western blot, we also demonstrated the reduction of GFAP and astrocyte marker protein levels in the brain of the 5xFAD-P group (cortex, 1.38 ± 0.07; hippocampus, 1.21 ± 0.10; Figure 3B,C) compared to that in the 5xFAD-V group (cortex, 4.52 ± 0.6, *p* < 0.0001; hippocampus, 1.67 ± 0.17, *p* < 0.05). Our results demonstrated that the PCO treatment attenuated astrogliosis in the 5xFAD mice.

**Figure 4:**
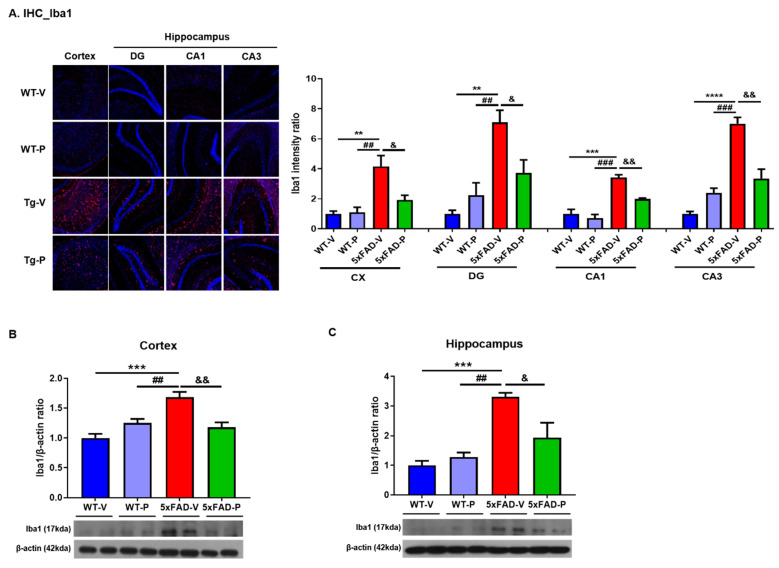
Cuban policosanol (PCO) treatment attenuated induced microgliosis in 5xFAD mice. To observe microglia alteration, (**A**) image and quantification of Iba1 immunofluorescence stain in the cortex (CX) and hippocampus (DG, CA1, and CA3). The Iba1 expression level in the 5xFAD-P group was significantly decreased compared to the 5xFAD-V group. Western blot was performed in both (**B**) the cortex and (**C**) the hippocampus. The policosanol treatment in the 5xFAD attenuated overexpression level of Iba1, which is a marker of microglia, compared to the 5xFAD-V group. Values are expressed as the mean ± SEM (*n* = 4 per group). ** *p* < 0.01, *** *p* < 0.001, and **** *p* < 0.0001 vs. WT-V group, ^##^ *p* < 0.01, and ^###^ *p* < 0.001 vs. WT-P group, ^&^ *p* < 0.05, and ^&&^ *p* < 0.01 vs. 5xFAD-V group. Statistical analysis between four groups was performed using a one-way analysis of variance, followed by Turkey’s post hoc test.

Next, we performed immunofluorescence staining and Western blotting using the ionized calcium-binding adaptor molecule 1 (Iba-1) antibody, a microglial marker, in the brains of the mice (Figure 4). In the brains of the 5xFAD-V mice (cortex, 4.17 ± 0.72, DG, 7.10 ± 0.81; CA1, 3.42 ± 0.19; CA3, 7.01 ± 0.42), the number of Iba-1 positive brain cells in the cortex and hippocampus was significantly increased compared to the wild-type groups (WT-V, cortex, 1.00 ± 0.18, *p* < 0.01; DG, 1.00 ± 0.24, *p* < 0.01; CA1, 1.00 ± 0.30, *p* < 0.001; CA3, 1.00 ± 0.16, *p* < 0.0001; WT-P, cortex, 1.11 ± 0.33, *p* < 0.01; DG, 2.24 ± 0.83, *p* < 0.01; CA1, 0.70 ± 0.26, *p* < 0.001; CA3, 2.40 ± 0.31, *p* < 0.001; Figure 4A). However, PCO (cortex, 1.91 ± 0.32, *p* < 0.05, DG, 3.72 ± 0.88, *p* < 0.05; CA1, 1.99 ± 0.07, *p* < 0.01; CA3, 3.349 ± 0.63, *p* < 0.01) treatments significantly decreased the number of Iba-1 positive brain cells in the cortex and hippocampus compared to the 5xFAD-V group. In the Western blot analysis to detect the level of microglia, we found that the protein level of Iba1 in the cortex of the 5xFAD-P mice (1.18 ± 0.08, Figure 4B) was significantly reduced compared to the 5xFAD-V mice (1.683 ± 0.09, *p* < 0.01) and similar to the WT groups (WT-V, 1.00 ± 0.07; WT-P, 1.25 ± 0.07). In the hippocampus of the 5xFAD-V mice (3.31 ± 0.13, Figure 4C), the level of Iba1 was significantly increased compared to the WT groups (WT-V, 1.00 ± 0.15, *p* < 0.001; WT-P, 1.28 ± 0.15, *p* < 0.01). In the PCO-treated 5xFAD group (1.94 ± 0.50, *p* < 0.05), the Iba1 expression level was significantly lower than that in the 5xFAD-V group.

### 3.4. PCO Has the Antioxidant and Anti-Inflammatory Effect in the Brain of 5xFAD

**Figure 5:**
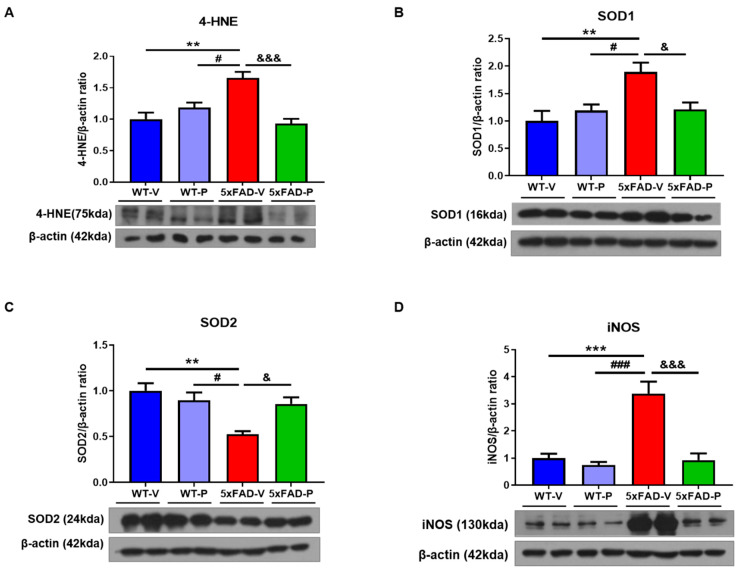
The policosanol showed an antioxidant effect on oxidative stress. In the Western blot, (**A**) the overexpression of 4-hydroxynonenal (4-HNE), which is a marker of oxidative stress, in the 5xFAD group was significantly attenuated by policosanol treatment. (B–D) Abnormal expressions of SOD1 (**B**), SOD2 (**C**), and iNOS (**D**) in the 5xFAD-P group recovered compared to the 5xFAD-V group. Values are expressed as the mean ± SEM (*n* = 4–5 per group). ** *p* < 0.01, and *** *p* < 0.001 vs. WT-V group, ^#^ *p* < 0.05, and ^###^ *p* < 0.001 vs. WT-P group, ^&^ *p* < 0.05, and ^&&&^ *p* < 0.001 vs. 5xFAD-V group. Statistical analysis between the four groups was performed using the one-way analysis of variance, followed by Turkey’s post hoc test.

Next, Western blots were used to determine the oxidative stress and inflammatory changes induced by altered Aβ and gliosis. As shown in Figure 5A, the protein level of 4-HNE in 5xFAD-V mice (1.65 ± 0.10) was significantly increased compared to that in the WT-V mice (1.00 ± 0.11, *p* < 0.01) or WT-P (1.18 ± 0.08, *p* < 0.05, Figure 5A) mice. The 5xFAD-P mice (0.93 ± 0.08, *p* < 0.001) showed a reduced 4-HNE level compared to the 5xFAD-V mice. SOD is the primary defense antioxidant that suppresses or prevents the formation of free radicals or reactive species in cells. Therefore, we investigated the level of SOD in the cortex of mice to confirm the effect of PCO on oxidative stress in the 5xFAD mice. The level of SOD1 in the 5xFAD-V mice (1.89 ± 0.17) increased compared to that in the WT mice (WT-V, 1.00 ± 0.18, *p* < 0.01; WT-P, 1.19 ± 0.11, *p* < 0.05; Figure 5B). The PCO treatment (1.21 ± 0.12, *p* < 0.05) significantly restored the protein level of SOD1 in the 5xFAD mice compared to that in the WT mice. However, the level of SOD2 protein in the 5xFAD-V (0.53 ± 0.03, Figure 5C) group significantly decreased compared to the WT groups (WT-V, 1.00 ± 0.08, *p* < 0.01; WT-P, 0.90 ± 0.09, *p* < 0.05). In the 5xFAD-P group, the protein level of SOD2 (1.21 ± 0.12, *p* < 0.05) recovered, similar to the WT group.

The nitric oxide radical (^•^NO) is a representative reactive species in oxidative stress. The expression level of inducible nitric oxide synthase in the 5xFAD-V mice (3.38 ± 0.45, Figure 5D) was significantly increased compared to that in the wild-type mice (WT-V, 1.00 ± 0.17, *p* < 0.001; WT-P, 0.74 ± 0.12, *p* < 0.001). PCO-treated 5xFAD mice (5xFAD-P, 0.93 ± 0.25, *p* < 0.001) showed a significant reduction in the iNOS expression level compared to the 5xFAD-V mice.

**Figure 6:**
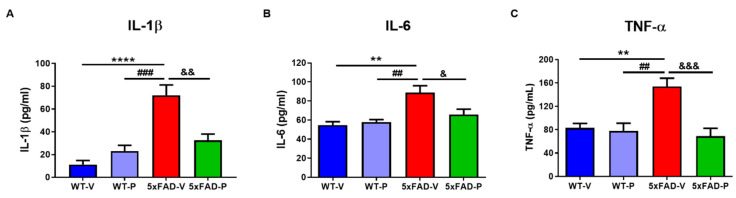
The policosanol treatment appeared as an anti-inflammatory effect. In the 5xFAD-P group, the level of pro-inflammatory cytokines such as IL-1β (**A**), IL-6 (**B**), and TNF-α (**C**) was significantly decreased compared to the 5xFAD-V group. Values are expressed as the mean ± SEM (*n* = 5–6 per group). ** *p* < 0.01, and **** *p* < 0.0001 vs. WT-V group, ^##^ *p* < 0.01, and ^###^ *p* < 0.001 vs. WT-P group, ^&^ *p* < 0.05, ^&&^ *p* < 0.01, and ^&&&^ *p* < 0.001 vs. 5xFAD-V group. Statistical analysis was performed using the one-way ANOVA, followed by Turkey’s post hoc test.

To investigate the effect of PCO treatment on the immune response, we measured the protein expression levels of pro-inflammatory cytokines such as IL-1β, IL-6, and TNF-α. In the cortex of the 6-month aged 5xFAD-V mice, IL-1β was significantly increased compared to the control group (11.32 ± 3.574 pg/mL, *p* < 0.0001, WT-V; 23.13 ± 4.987 pg/mL, *p* < 0.001, WT-P; Figure 6A). However, the PCO treatment in the 5xFAD mice (32.55 ± 5.527 pg/mL, *p* < 0.01) decreased the expression level of IL-1β compared to the 5xFAD-V mice. IL-6 in the 5xFAD-V mice (88.71 ± 7.375 pg/mL) was significantly increased compared to the cortex of the wild-type groups (54.79 ± 3.557 pg/mL, *p* < 0.01, WT-V; 57.87 ± 2.652 pg/mL, *p* < 0.01, WT-P; Figure 6B). However, the IL-6 expression level in the PCO-treated 5xFAD mice (65.83 ± 5.62 pg/mL, *p* < 0.05) significantly decreased compared to the 5xFAD-V mice. Moreover, TNF-α in the cortex of the 5xFAD-V mice (154.1 ± 13.97 pg/mL) was significantly upregulated compared to the age-matched control group (83.26 ± 7.412 pg/mL, *p* < 0.01, WT-V; 77.96 ± 13.04 pg/mL, *p* < 0.01, WT-P; Figure 6C). However, the TNF-α protein level in the 5xFAD-P group (68.92 ± 13.44 pg/mL, *p* < 0.001) significantly decreased, similar to that in the wild-type group. As a result, we confirmed that the long-term PCO treatment attenuated abnormal inflammatory response-induced amyloid plaques in the 5xFAD mice.

### 3.5. PCO Treatment Attenuates Synaptic loss in 5xFAD Mice

**Figure 7:**
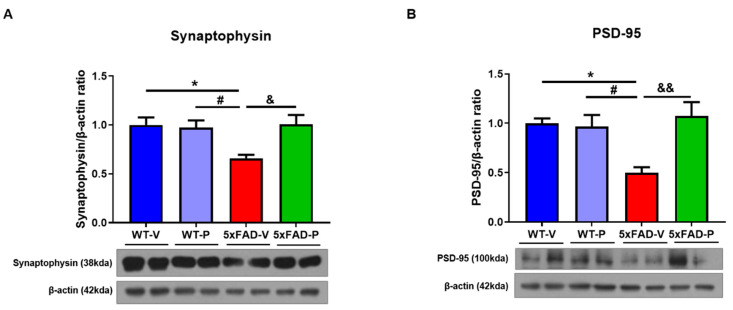
The policosanol treatment inhibited the synaptic loss in the hippocampus of the 5xFAD mice. (**A**) Synaptophysin, which is a presynaptic marker, and (**B**) PSD-95, which is a postsynaptic marker were recovered by policosanol administration in the 5xFAD-P group. Values are expressed as the mean ± standard error of the mean (*n* = 4 per group). * *p* < 0.05 vs. WT-V group, ^#^ *p* < 0.05 vs. WT-P group, ^&^ *p* < 0.05, and ^&&^ *p* < 0.01 vs. 5xFAD-V group. Statistical analysis between the four groups was performed using the one-way analysis of variance, followed by Turkey’s post hoc test.

In the 5xFAD mice, synaptic loss, including the presynapse and the post-synapse, was observed in the 6-month mice [31]. Therefore, we assessed the presynaptic marker synaptophysin, and postsynaptic marker PSD-95 in the cortex of the mice. The expression level of the synaptophysin in the brain of the 5xFAD-V mice (0.659 ± 0.035) was significantly decreased compared to that in the wild-type mice (WT-V, 1.00 ± 0.08, *p* < 0.05; WT-P, 0.97 ± 0.07, *p* < 0.05; Figure 7A). Interestingly, the protein expression level of synaptophysin in the 5xFAD-P group (1.01 ± 0.097, *p* < 0.05) recovered compared to that in the 5xFAD-V group. In addition, we also confirmed PSD-95, a postsynaptic marker, in the cortex of mice. The protein level of PSD-95 in the 5xFAD-V group (0.499 ± 0.057) was significantly lower than that of the control group (WT-V, 1.00 ± 0.049, *p* < 0.05; WT-P, 0.967 ± 0.117, *p* < 0.05; Figure 7B). In the PCO treatment of the 5xFAD mice (1.07 ± 0.14, *p* < 0.01), the PSD-95 expression level recovered similar to that in the wild-type group.

## 4. Discussion

This study demonstrates that long-term treatment with PCO has a protective effect on AD progression. In detail, we showed that the PCO treatment of 5xFAD mice for 4-months attenuated memory impairment, amyloid plaque formation, and gliosis, including the astrocytes and microglia, in the brain. Together with these results, we demonstrated that PCO consumption reduced lipid peroxidation and aberrant SOD proteins, thereby reducing the inflammatory response and eventually normalizing synaptic loss in the 5xFAD mice.

PCO derived from the wax component of plants is a long-chain aliphatic alcohol compound comprising eight kinds of sugar cane originating from aliphatic alcohols [26]. Previous studies have reported that PCO intake improves abnormal lipoprotein levels or cardiovascular disease [26,32]. It also showed enhancement of HDL-C functionality, reduction of total cholesterol [31], and LDL-cholesterol (LDL-C) in human blood samples [28,33]. These improvements imply that the PCO is a potent natural antioxidant [34]. In addition, PCO treatment has been reported to be effective in ameliorating neuronal damage or plasma oxidative stress in a stroke animal model [35]. Dyslipidemia is a common risk factor associated with degenerative brain diseases such as AD [36,37]. For example, several studies have suggested that a circulating high level of LDL-C is associated with cognitive decline or early-onset AD [38,39]. Furthermore, high HDL-C levels are associated with a decreased risk of AD in elderly individuals [40]. Although the specific mechanism has not been fully elucidated, apolipoprotein A-I (apoA-I), which is the most abundant lipoprotein in HDL, and discoidal HDL, have a protective effect in AD pathology, including amelioration of Aβ deposition and memory reduction as well as the antioxidant effect in preclinical studies [41,42,43,44]. ApoA-I and discoidal HDL was known to delay Aβ fibrillization in the brain through access via the blood-cerebrospinal fluid barrier at the choroid plexus [45]. It is well known that apoA-I and HDL can inhibit amyloid plaque aggregation and remove Aβ from the brain [45,46]. Moreover, in our study, PCO administration inhibited the formation of Aβ plaques, delaying AD pathology progression in 5xFAD mice.

Early-onset AD is mainly familial and is caused by mutations in the gene encoding APP, PS-1 (PSEN1), and PS-2 (PSEN2) [1]. Since 5xFAD mice include five familial AD (FAD) mutations consisting of amyloid protein precursor (APP) and presenilin-1 (PSEN1), they showed Aβ plaques and cognitive impairment in the early stage [47]. In our results, we confirmed the increased Aβ plaques and high protein levels of Aβ_1–42_ in the 5xFAD mice of 6-months (Figure 2). In the early stage of AD, amyloid plaques appear, and the reactivation or activation of glial cells, such as astrocytes and microglia, are located near the plaques [12]. Astrocytes and microglia play a major role in the Aβ clearance and degradation through the production of proteases capable of hydrolyzing Aβ at different cleavage sites or accelerating Aβ exit to the blood circulation [12,48]. Gliosis following Aβ *plaque* stimulation releases a variety of pro-inflammatory cytokines, including IL-1*β*, IL-6, and TNF-α [49], resulting in a persistent inflammatory response [11]. However, Aβ exposure induces astrocytic cell death as well as neuronal cell death through upregulation of the inflammatory cytokines and nitric oxide release [50].

Activated microglia are also associated with the acceleration of neuronal dysfunction after the production of neurotoxic cytokines, chemokines, and reactive oxygen and nitrogen species [51,52]. The treatment agent which affects neuroinflammation has been focused as a new pharmacological tool for neurodegenerative disease [53]. In our results, the PCO treatment attenuated the abnormal astrocytes and microglia in the brain of the mice, as well as the inflammatory response (Figure 3, Figure 4 and Figure 6). Long-term PCO treatment considerably inhibited the formation of amyloid plaques composed of Aβ_1–42_, thereby suppressing gliosis and restoring pro-inflammatory cytokines.

The formation of amyloid plaques composed of amyloid-beta (Aβ) peptides is initiated and enhanced by oxidative stress and an imbalance between the oxidants and antioxidants [54]. Previous studies demonstrated that, in patients with AD, oxidative stress occurred in the brain region of abundant Aβ_1–42_, which led to lipid peroxidation [55]. Moreover, a higher concentration of iron (Fe) is found in the brains of patients with AD, such as the hippocampus and the parietal cortex [56], which results in damage to the neuronal cell membrane and cell death via lipid peroxidation [57]. Lipid peroxidation is a major target of neurodegenerative diseases since the brain, which consumes nearly 20% of inspired oxygen and comprises high levels of polyunsaturated fatty acids (PUFAs), is very susceptible to oxidative stress [58]. In particular, the attack of free radicals on the PUFAs in the membrane phospholipids results in the formation of reactive aldehydes such as 4-HNE and malondialdehyde [59]. Although the normal brain is protected from oxidative stress by antioxidant or free radical scavenging, impairment of these mechanisms results in excessive lipid peroxidation [60,61], and DNA oxidation [62], and protein oxidation [63] in the brain of a patient with AD. Numerous studies have demonstrated that the 4-HNE level, a lipid peroxidation marker, was increased in patients and animal models of AD [64,65,66]. The 4-HNE is highly neurotoxic, affects the histological alteration in the AD brain, and correlates with increased neuronal apoptosis [64,67]. The accumulation of free radical damage alters the activity or expression of antioxidants, including SOD or catalase, in the brains of patients with AD [68,69]. Although it is controversial for SOD expression levels in the AD brain, some research has shown that expression of SOD1 (CuZn-SOD), which is localized in the cytosol, was increased in clinical and preclinical studies. [23,70,71]. In contrast, SOD2 (Mn-SOD), which is the main superoxide scavenger in the mitochondria, exacerbates oxidative stress and the pathology of AD [72,73]. After 4-months of PCO treatment, excessive lipid peroxide (4-HNE) with abnormal SOD1 and SOD2 in the 5xFAD mice recovered (Figure 5), PCO is considered to have inhibited the oxidative damage induced by amyloid plaques. Prolonged oxidative stress induced by Aβ plaques is known to result in synaptic loss associated with memory impairment [74]. However, long-term administration of PCO suppressed the formation of amyloid plaques, resulting in anti-inflammatory and anti-oxidative effects. Consequently, it was confirmed to restore damaged synaptic proteins such as synaptophysin and PSD-95 (Figure 7) and memory impairment (Figure 1) in policosanol-treated 5xFAD mice.

This study had certain limitations. First, we evaluated the protective efficiency of PCO in the 5xFAD male mice. Since there is evidence of sex-specific patterns and sex differences in AD [75], the PCO effects in female mice need to be confirmed. Together with previous studies that examined the effect of PCO on ameliorating dyslipidemia, we confirmed the inhibition of amyloid plaque formation. Second, following the blood-brain barrier, studies on the clear mechanism of PCO action on the regulation between apoA-I and amyloid pathology should be performed. Finally, based on the report on the increase of HDL-C and improvement of HDL function with PCO treatment, we need to determine the protective efficacy of PCO against vascular dementia, the second most common cause of dementia, which could be prevented by upregulation of HDL-C level [40,45].

Taken together, there is a possibility that the treatment with PCO causes elevation of HDL-C and enhancement of HDL functionality to induce the reduction of oxidized species (4-HNE and iNOS), inflammatory cytokines (IL-1β, IL-6, and TNF-α), and amyloid plaque size. The current results could explain the relationship between higher serum HDL-C in middle age and the lower incidence of dementia in late age in a previous human study [76].

In summary, we demonstrated that PCO has antioxidant and anti-inflammatory effects by inhibiting the formation of Aβ plaques in the brain, and could be an effective supplement in delaying or preventing AD progression.

## Data Availability

The data presented in this study are available in the article.

## References

[1] MartinPrince A.C.-H. Martin Knapp, Maëlenn Guerchet, Maria Karagiannidou. World Alzheimer Report 2016: Impoving healthcare for people living with dementia: Coverage, quality and costs now and in the future. http://www.alz.co.uk/.

[2] Serrano-Pozo A., Frosch M.P., Masliah E., Hyman B.T. (2011). Neuropathological alterations in Alzheimer disease. Cold Spring Harb. Perspect Med..

[3] Haque R.U., Levey A.I. (2019). Alzheimer’s disease: A clinical perspective and future nonhuman primate research opportunities. Proc. Natl. Acad. Sci. USA.

[4] Campora M., Francesconi V., Schenone S., Tasso B., Tonelli M. (2021). Journey on Naphthoquinone and Anthraquinone Derivatives: New Insights in Alzheimer’s Disease. Pharmaceuticals.

[5] Chen G.F., Xu T.H., Yan Y., Zhou Y.R., Jiang Y., Melcher K., Xu H.E. (2017). Amyloid beta: Structure, biology and structure-based therapeutic development. Acta Pharmacol. Sin..

[6] Bloom G.S. (2014). Amyloid-beta and tau: The trigger and bullet in Alzheimer disease pathogenesis. JAMA Neurol..

[7] Selkoe D.J. (2001). Alzheimer’s disease: Genes, proteins, and therapy. Physiol. Rev..

[8] Iwatsubo T., Odaka A., Suzuki N., Mizusawa H., Nukina N., Ihara Y. (1994). Visualization of A beta 42(43) and A beta 40 in senile plaques with end-specific A beta monoclonals: Evidence that an initially deposited species is A beta 42(43). Neuron.

[9] Guo T., Zhang D., Zeng Y., Huang T.Y., Xu H., Zhao Y. (2020). Molecular and cellular mechanisms underlying the pathogenesis of Alzheimer’s disease. Mol. Neurodegener..

[10] Selkoe D.J., Hardy J. (2016). The amyloid hypothesis of Alzheimer’s disease at 25 years. EMBO Mol. Med..

[11] Kinney J.W., Bemiller S.M., Murtishaw A.S., Leisgang A.M., Salazar A.M., Lamb B.T. (2018). Inflammation as a central mechanism in Alzheimer’s disease. Alzheimers Dement..

[12] Olabarria M., Noristani H.N., Verkhratsky A., Rodriguez J.J. (2010). Concomitant astroglial atrophy and astrogliosis in a triple transgenic animal model of Alzheimer’s disease. Glia.

[13] Stalder M., Phinney A., Probst A., Sommer B., Staufenbiel M., Jucker M. (1999). Association of microglia with amyloid plaques in brains of APP23 transgenic mice. Am. J. Pathol..

[14] Swardfager W., Lanctot K., Rothenburg L., Wong A., Cappell J., Herrmann N. (2010). A meta-analysis of cytokines in Alzheimer’s disease. Biol. Psychiatry.

[15] Frost G.R., Li Y.M. (2017). The role of astrocytes in amyloid production and Alzheimer’s disease. Open Biol..

[16] Parajuli B., Sonobe Y., Horiuchi H., Takeuchi H., Mizuno T., Suzumura A. (2013). Oligomeric amyloid beta induces IL-1beta processing via production of ROS: Implication in Alzheimer’s disease. Cell Death Dis..

[17] Markesbery W.R. (1999). The role of oxidative stress in Alzheimer disease. Arch. Neurol..

[18] Persson T., Popescu B.O., Cedazo-Minguez A. (2014). Oxidative stress in Alzheimer’s disease: Why did antioxidant therapy fail?. Oxid. Med. Cell Longev..

[19] Chauhan V., Chauhan A. (2006). Oxidative stress in Alzheimer’s disease. Pathophysiology.

[20] Wang W., Zhao F., Ma X., Perry G., Zhu X. (2020). Mitochondria dysfunction in the pathogenesis of Alzheimer’s disease: Recent advances. Mol. Neurodegener..

[21] Sayre L.M., Zelasko D.A., Harris P.L., Perry G., Salomon R.G., Smith M.A. (1997). 4-Hydroxynonenal-derived advanced lipid peroxidation end products are increased in Alzheimer’s disease. J. Neurochem..

[22] Mark R.J., Lovell M.A., Markesbery W.R., Uchida K., Mattson M.P. (1997). A role for 4-hydroxynonenal, an aldehydic product of lipid peroxidation, in disruption of ion homeostasis and neuronal death induced by amyloid beta-peptide. J. Neurochem..

[23] Zemlan F.P., Thienhaus O.J., Bosmann H.B. (1989). Superoxide dismutase activity in Alzheimer’s disease: Possible mechanism for paired helical filament formation. Brain Res..

[24] Murakami K., Murata N., Noda Y., Tahara S., Kaneko T., Kinoshita N., Hatsuta H., Murayama S., Barnham K.J., Irie K. (2011). SOD1 (copper/zinc superoxide dismutase) deficiency drives amyloid beta protein oligomerization and memory loss in mouse model of Alzheimer disease. J. Biol. Chem..

[25] Gong J., Qin X., Yuan F., Hu M., Chen G., Fang K., Wang D., Jiang S., Li J., Zhao Y. (2018). Efficacy and safety of sugarcane policosanol on dyslipidemia: A meta-analysis of randomized controlled trials. Mol. Nutr. Food Res..

[26] Janikula M. (2002). Policosanol: A new treatment for cardiovascular disease?. Altern. Med. Rev..

[27] Park H.J., Yadav D., Jeong D.J., Kim S.J., Bae M.A., Kim J.R., Cho K.H. (2019). Short-Term Consumption of Cuban Policosanol Lowers Aortic and Peripheral Blood Pressure and Ameliorates Serum Lipid Parameters in Healthy Korean Participants: Randomized, Double-Blinded, and Placebo-Controlled Study. Int. J. Environ. Res. Public Health.

[28] Kim S.J., Yadav D., Park H.J., Kim J.R., Cho K.H. (2018). Long-Term Consumption of Cuban Policosanol Lowers Central and Brachial Blood Pressure and Improves Lipid Profile With Enhancement of Lipoprotein Properties in Healthy Korean Participants. Front. Physiol..

[29] Lee E.Y., Yoo J.A., Lim S.M., Cho K.H. (2016). Anti-Aging and Tissue Regeneration Ability of Policosanol Along with Lipid-Lowering Effect in Hyperlipidemic Zebrafish via Enhancement of High-Density Lipoprotein Functionality. Rejuvenation Res..

[30] Kang S., Kim J., Chang K.A. (2021). Spatial memory deficiency early in 6xTg Alzheimer’s disease mouse model. Sci. Rep..

[31] Crouzin N., Baranger K., Cavalier M., Marchalant Y., Cohen-Solal C., Roman F.S., Khrestchatisky M., Rivera S., Feron F., Vignes M. (2013). Area-specific alterations of synaptic plasticity in the 5XFAD mouse model of Alzheimer’s disease: Dissociation between somatosensory cortex and hippocampus. PLoS ONE.

[32] Gouni-Berthold I., Berthold H.K. (2002). Policosanol: Clinical pharmacology and therapeutic significance of a new lipid-lowering agent. Am. Heart J..

[33] Cho K.H., Kim S.J., Yadav D., Kim J.Y., Kim J.R. (2018). Consumption of Cuban Policosanol Improves Blood Pressure and Lipid Profile via Enhancement of HDL Functionality in Healthy Women Subjects: Randomized, Double-Blinded, and Placebo-Controlled Study. Oxid. Med. Cell Longev..

[34] Kim J.Y., Kim S.M., Kim S.J., Lee E.Y., Kim J.R., Cho K.H. (2017). Consumption of policosanol enhances HDL functionality via CETP inhibition and reduces blood pressure and visceral fat in young and middle-aged subjects. Int. J. Mol. Med..

[35] Molina V., Ravelo Y., Noa M., Mas R., Perez Y., Oyarzabal A., Mendoza N., Valle M., Jimenez S., Sanchez J. (2013). Therapeutic Effects of Policosanol and Atorvastatin against Global Brain Ischaemia-Reperfusion Injury in Gerbils. Indian J. Pharm Sci..

[36] Alzheimer’s A. (2016). 2016 Alzheimer’s disease facts and figures. Alzheimers Dement..

[37] Marsillach J., Adorni M.P., Zimetti F., Papotti B., Zuliani G., Cervellati C. (2020). HDL Proteome and Alzheimer’s Disease: Evidence of a Link. Antioxidants.

[38] Wingo T.S., Cutler D.J., Wingo A.P., Le N.A., Rabinovici G.D., Miller B.L., Lah J.J., Levey A.I. (2019). Association of Early-Onset Alzheimer Disease With Elevated Low-Density Lipoprotein Cholesterol Levels and Rare Genetic Coding Variants of APOB. JAMA Neurol..

[39] Zhou Z., Liang Y., Zhang X., Xu J., Lin J., Zhang R., Kang K., Liu C., Zhao C., Zhao M. (2020). Low-Density Lipoprotein Cholesterol and Alzheimer’s Disease: A Systematic Review and Meta-Analysis. Front. Aging Neurosci..

[40] Reitz C., Tang M.X., Schupf N., Manly J.J., Mayeux R., Luchsinger J.A. (2010). Association of higher levels of high-density lipoprotein cholesterol in elderly individuals and lower risk of late-onset Alzheimer disease. Arch. Neurol..

[41] Lewis T.L., Cao D., Lu H., Mans R.A., Su Y.R., Jungbauer L., Linton M.F., Fazio S., LaDu M.J., Li L. (2010). Overexpression of human apolipoprotein A-I preserves cognitive function and attenuates neuroinflammation and cerebral amyloid angiopathy in a mouse model of Alzheimer disease. J. Biol. Chem..

[42] Dal Magro R., Simonelli S., Cox A., Formicola B., Corti R., Cassina V., Nardo L., Mantegazza F., Salerno D., Grasso G. (2019). The Extent of Human Apolipoprotein A-I Lipidation Strongly Affects the beta-Amyloid Efflux Across the Blood-Brain Barrier in vitro. Front. Neurosci..

[43] Paterno R., Ruocco A., Postiglione A., Hubsch A., Andresen I., Lang M.G. (2004). Reconstituted high-density lipoprotein exhibits neuroprotection in two rat models of stroke. Cerebrovasc. Dis..

[44] Button E.B., Boyce G.K., Wilkinson A., Stukas S., Hayat A., Fan J., Wadsworth B.J., Robert J., Martens K.M., Wellington C.L. (2019). ApoA-I deficiency increases cortical amyloid deposition, cerebral amyloid angiopathy, cortical and hippocampal astrogliosis, and amyloid-associated astrocyte reactivity in APP/PS1 mice. Alzheimers Res. Ther..

[45] Button E.B., Robert J., Caffrey T.M., Fan J., Zhao W., Wellington C.L. (2019). HDL from an Alzheimer’s disease perspective. Curr. Opin. Lipidol..

[46] Zimetti F., Adorni M.P., Marsillach J., Marchi C., Trentini A., Valacchi G., Cervellati C. (2021). Connection between the Altered HDL Antioxidant and Anti-Inflammatory Properties and the Risk to Develop Alzheimer’s Disease: A Narrative Review. Oxid. Med. Cell Longev..

[47] Sasaguri H., Nilsson P., Hashimoto S., Nagata K., Saito T., De Strooper B., Hardy J., Vassar R., Winblad B., Saido T.C. (2017). APP mouse models for Alzheimer’s disease preclinical studies. EMBO J..

[48] Ries M., Sastre M. (2016). Mechanisms of Abeta Clearance and Degradation by Glial Cells. Front. Aging Neurosci..

[49] Minter M.R., Taylor J.M., Crack P.J. (2016). The contribution of neuroinflammation to amyloid toxicity in Alzheimer’s disease. J. Neurochem..

[50] Hu J., Akama K.T., Krafft G.A., Chromy B.A., Van Eldik L.J. (1998). Amyloid-beta peptide activates cultured astrocytes: Morphological alterations, cytokine induction and nitric oxide release. Brain Res..

[51] Fuhrmann M., Bittner T., Jung C.K., Burgold S., Page R.M., Mitteregger G., Haass C., LaFerla F.M., Kretzschmar H., Herms J. (2010). Microglial Cx3cr1 knockout prevents neuron loss in a mouse model of Alzheimer’s disease. Nat. Neurosci..

[52] Mandrekar-Colucci S., Landreth G.E. (2010). Microglia and inflammation in Alzheimer’s disease. CNS Neurol. Disord. Drug Targets.

[53] Villa V., Thellung S., Bajetto A., Gatta E., Robello M., Novelli F., Tasso B., Tonelli M., Florio T. (2016). Novel celecoxib analogues inhibit glial production of prostaglandin E2, nitric oxide, and oxygen radicals reverting the neuroinflammatory responses induced by misfolded prion protein fragment 90-231 or lipopolysaccharide. Pharmacol. Res..

[54] Huang W.J., Zhang X., Chen W.W. (2016). Role of oxidative stress in Alzheimer’s disease. Biomed. Rep..

[55] Butterfield D.A., Boyd-Kimball D. (2018). Oxidative Stress, Amyloid-beta Peptide, and Altered Key Molecular Pathways in the Pathogenesis and Progression of Alzheimer’s Disease. J. Alzheimers Dis..

[56] Qin Y., Zhu W., Zhan C., Zhao L., Wang J., Tian Q., Wang W. (2011). Investigation on positive correlation of increased brain iron deposition with cognitive impairment in Alzheimer disease by using quantitative MR R2’ mapping. J. Huazhong Univ. Sci. Technolog. Med. Sci..

[57] Nizzari M., Thellung S., Corsaro A., Villa V., Pagano A., Porcile C., Russo C., Florio T. (2012). Neurodegeneration in Alzheimer disease: Role of amyloid precursor protein and presenilin 1 intracellular signaling. J. Toxicol..

[58] Sultana R., Perluigi M., Butterfield D.A. (2013). Lipid peroxidation triggers neurodegeneration: A redox proteomics view into the Alzheimer disease brain. Free Radic Biol. Med..

[59] Montine T.J., Neely M.D., Quinn J.F., Beal M.F., Markesbery W.R., Roberts L.J., Morrow J.D. (2002). Lipid peroxidation in aging brain and Alzheimer’s disease. Free Radic. Biol. Med..

[60] Pratico D., Uryu K., Leight S., Trojanoswki J.Q., Lee V.M. (2001). Increased lipid peroxidation precedes amyloid plaque formation in an animal model of Alzheimer amyloidosis. J. Neurosci..

[61] Butterfield D.A., Lauderback C.M. (2002). Lipid peroxidation and protein oxidation in Alzheimer’s disease brain: Potential causes and consequences involving amyloid beta-peptide-associated free radical oxidative stress. Free Radic Biol. Med..

[62] Mecocci P., MacGarvey U., Beal M.F. (1994). Oxidative damage to mitochondrial DNA is increased in Alzheimer’s disease. Ann. Neurol..

[63] Aksenov M.Y., Aksenova M.V., Butterfield D.A., Geddes J.W., Markesbery W.R. (2001). Protein oxidation in the brain in Alzheimer’s disease. Neuroscience.

[64] Markesbery W.R., Lovell M.A. (1998). Four-hydroxynonenal, a product of lipid peroxidation, is increased in the brain in Alzheimer’s disease. Neurobiol. Aging.

[65] McGrath L.T., McGleenon B.M., Brennan S., McColl D., Mc I.S., Passmore A.P. (2001). Increased oxidative stress in Alzheimer’s disease as assessed with 4-hydroxynonenal but not malondialdehyde. QJM.

[66] Lovell M.A., Xie C., Markesbery W.R. (2001). Acrolein is increased in Alzheimer’s disease brain and is toxic to primary hippocampal cultures. Neurobiol. Aging.

[67] Shoeb M., Ansari N.H., Srivastava S.K., Ramana K.V. (2014). 4-Hydroxynonenal in the pathogenesis and progression of human diseases. Curr. Med. Chem..

[68] Marcus D.L., Thomas C., Rodriguez C., Simberkoff K., Tsai J.S., Strafaci J.A., Freedman M.L. (1998). Increased peroxidation and reduced antioxidant enzyme activity in Alzheimer’s disease. Exp. Neurol..

[69] Zhao Y., Zhao B. (2013). Oxidative stress and the pathogenesis of Alzheimer’s disease. Oxid Med. Cell Longev..

[70] Choi J., Rees H.D., Weintraub S.T., Levey A.I., Chin L.S., Li L. (2005). Oxidative modifications and aggregation of Cu,Zn-superoxide dismutase associated with Alzheimer and Parkinson diseases. J. Biol. Chem..

[71] Grinan-Ferre C., Sarroca S., Ivanova A., Puigoriol-Illamola D., Aguado F., Camins A., Sanfeliu C., Pallas M. (2016). Epigenetic mechanisms underlying cognitive impairment and Alzheimer disease hallmarks in 5XFAD mice. Aging.

[72] Esposito L., Raber J., Kekonius L., Yan F., Yu G.Q., Bien-Ly N., Puolivali J., Scearce-Levie K., Masliah E., Mucke L. (2006). Reduction in mitochondrial superoxide dismutase modulates Alzheimer’s disease-like pathology and accelerates the onset of behavioral changes in human amyloid precursor protein transgenic mice. J. Neurosci..

[73] Lee H.P., Pancholi N., Esposito L., Previll L.A., Wang X., Zhu X., Smith M.A., Lee H.G. (2012). Early induction of oxidative stress in mouse model of Alzheimer disease with reduced mitochondrial superoxide dismutase activity. PLoS ONE.

[74] Tonnies E., Trushina E. (2017). Oxidative Stress, Synaptic Dysfunction, and Alzheimer’s Disease. J. Alzheimers Dis..

[75] Ferretti M.T., Iulita M.F., Cavedo E., Chiesa P.A., Schumacher Dimech A., Santuccione Chadha A., Baracchi F., Girouard H., Misoch S., Giacobini E. (2018). Sex differences in Alzheimer disease—the gateway to precision medicine. Nat. Rev. Neurol..

[76] Svensson T., Sawada N., Mimura M., Nozaki S., Shikimoto R., Tsugane S. (2019). The association between midlife serum high-density lipoprotein and mild cognitive impairment and dementia after 19 years of follow-up. Transl. Psychiatry.

